# Consumer Privacy Protection With the Growth of AI-Empowered Online Shopping Based on the Evolutionary Game Model

**DOI:** 10.3389/fpubh.2021.705777

**Published:** 2021-07-07

**Authors:** Su Wang, Zhuo Chen, Yi Xiao, Chunyu Lin

**Affiliations:** ^1^School of Economics, Ocean University of China, Qingdao, China; ^2^School of Innovation and Entrepreneurship, Shandong University, Jimo, China; ^3^Ocean College, Zhejiang University, Zhoushan, China

**Keywords:** social distancing, online shopping, privacy protection, evolutionary game, influencing mechanism

## Abstract

Social distancing due to the COVID-19 pandemic has driven some consumers to online shopping, and concerns about pandemic risks and personal hygiene have increased the demand for e-commerce. Providing personalized recommendations seems quite profitable for e-commerce platforms, and consumers also benefit from personalized content with the advancement of AI technologies. However, this possible win-win situation is marred by the increase in consumers' privacy concerns. Technical solutions have been widely studied to protect consumer privacy, while few analyses have been conducted from the perspective of psychological and behavioral implications. In this paper, an evolutionary game model of privacy protection between e-commerce platforms and consumers is established to determine the mechanisms by which various factors exert influence, and evolutionary stable strategies are obtained from equilibrium points. Then, the strategy selections are simulated with MATLAB 2020 software. Based on the results, the following conclusions are drawn: (1) the application of AI technologies in e-commerce will fundamentally benefit consumers, which makes them actively share personal information with e-commerce platforms with incentives for generous rewards; (2) it is profitable for e-commerce platforms to conduct data mining by improving the ability to use AI technologies and making efforts to reduce technical costs; and (3) regulators should improve the level of supervision instead of imposing a large penalty to enhance consumer trust, which could effectively increase the profits of e-commerce platforms and protect consumers' privacy.

## Introduction

As an important and effective measure to combat the COVID-19 pandemic, social distancing has encouraged consumers to use online shopping, which increases the demand for e-commerce (1–4). Continued social distancing results from the risk of adverse consequences of the COVID-19 pandemic, and increased concerns over personal hygiene will promote continued online shopping, reinforcing long-term changes in consumption patterns (5). E-commerce transactions involve transferring information online (6). Benefiting from the rapid growth of consumer data, the improved accuracy of artificial intelligence algorithms, the increased operational capability of computers, and advances in AI technologies such as machine learning, computer vision, and natural language processing, private information is collected, stored and processed on an unprecedented scale (7). This enables e-commerce platforms to accurately forecast customer demand. However, information asymmetry, externalities and commitment concerns can all be exacerbated by AI (8), and e-commerce challenges appear, including cybersecurity concerns and difficulties in gaining consumer trust (9). Consumers are paying increasing attention to their private information and becoming increasingly concerned about how data on shopping and transactions are collected and used (10–13). Thus, it is necessary to explore the relationship between e-commerce platforms and consumers in terms of privacy protection and accordingly take effective measures to balance the interests of both sides.

Studies on privacy protection mainly focus on ensuring the security of private information in the process of customer use. Technical management research is the main perspective and can be categorized into three aspects: an anonymity framework for privacy protection [e.g., (14–16)], privacy access control [e.g., (17–19)], and differential privacy (20, 21). Overall, most of the studies are general, and privacy preservation in medical care and online social networks (OSNs) are receiving increasing attention. Online shopping has received little attention, and there are few studies investigating privacy protection from the perspective of psychological and behavioral implications. Game theory is considered one of the most promising methodologies to investigate participants' incentives, responses, and behaviors (22). Many scholars investigate the behaviors of participants under the assumption of perfect rationality, which ignores the limitations of game players in reality due to limited cognitive abilities and complex decision-making situations [e.g., (23–25)]. Some scholars have introduced evolutionary game theory to overcome such limitations in recent years, and privacy protection in fields such as social networks (26) and e-commerce (27) and technologies such as the Internet of things (IOT) and cloud services have been the primary subjects investigated [e.g., (22, 28)].

In this paper, evolutionary game theory is applied to privacy protection in online shopping due to the rapidly increasing application of AI technologies in e-commerce platforms. An evolutionary game model of privacy protection between consumers and e-commerce platforms based on bounded rationality is established in Section Materials and Methods. In Section Evolutionary Game Analysis, the game mechanism is analyzed, and evolutionary stable strategies (ESSs) are obtained from equilibrium points. Section Simulation Results and Analysis presents the results of simulation experiments, which can directly display the decision-making results and the mechanisms by which various factors exert influence. Finally, targeted and practical measures and suggestions are proposed.

## Materials and Methods

### Evolutionary Game Theory

Compared with non-cooperative game theory, evolutionary game theory abandons the assumption of perfect rationality and incorporates a formation mechanism from individual behavior to group behavior and various factors involved in the evolutionary game model. Evolutionary game theory is based on the bounded rationality of group players and on the premise of incomplete information. The behavioral rules and strategies of game participants are modified and improved through continuous learning and imitation in the process of evolution, and decisions are made according to the benefits delivered by the strategies under comparison.

The evolutionary game process includes two stages: selection and mutation, which could be applied to privacy protection in online shopping. Selection means that a strategy that can deliver substantial benefits would be selected by more consumers and e-commerce platforms based on the benefit maximization principle. Mutation means that a consumer or e-commerce platform will choose strategies different from those adopted by the group due to random factors such as the introduction of third-party regulators. Mutation is often treated as a crucial aspect of the process of evolution since it can change the strategy choices of participants and eventually affect the equilibrium of the system.

As a tool of theoretical analysis that combines game theory with a dynamic evolution process, evolutionary games can well explain decision-making behaviors that change over time. Therefore, this study uses evolutionary game theory to explore the relationship between consumer privacy concerns and the behavioral decision-making of e-commerce platforms. Consumers and e-commerce platforms are boundedly rational, and their decisions are independent and made under asymmetric information; that is, the two players do not know each other's strategies at a given stage of the game, and their strategy choices are affected by the results of the previous stage.

### Problem Description

E-commerce platforms and all registered consumers using e-commerce platforms are the two players in the evolutionary game model of privacy protection. Based on previous studies on consumer privacy (29–32) and AI technologies in e-commerce (33), private information in this study includes demographic information (e.g., age, nationality, gender), personal financial information, personal identity information (e.g., username, gender, occupation, address), online shopping behavior (e.g., browsing history, browsing time, shopping habits), etc.

Consumers are normally required to allow access to personal information when they register an account for online shopping on e-commerce platforms. Some online consumers who value personalized content are willing to share personal data in return for price discounts, products or services, a small incentive or personalization (34–39), while others will not share information due to privacy concerns. Thus, the privacy protection strategy of consumers concerns whether to share private information.

Providing personalization is highly profitable for e-commerce platforms (40) since the mining of personal data can help e-commerce platforms better understand consumer preferences and promote marketing transformation. Therefore, e-commerce platforms may conduct data mining when consumers choose to share personal information or conduct illegal data mining to pursue their economic interests despite lacking consumers' permission. However, e-commerce platforms may not engage in data mining due to the imperfection of information shared by consumers or the low use value of such information. Based on the analysis above, the behavioral strategy of e-commerce platforms concerns whether to engage in mining.

History suggests that industry self-regulation may not occur absent the threat of government regulation in terms of privacy protection (41). Third-party regulators need to supervise the privacy protection measures of e-commerce platforms due to the information asymmetry between consumers and e-commerce platforms (8). Hence, third-party supervision is considered in this paper.

### Assumptions

#### Consumers

Consumers obtain fixed benefits (such as expanding the range of goods to choose, improving the convenience of price comparison, reducing transportation costs, etc.) regardless of whether they choose to share personal information when shopping on e-commerce platforms. However, by “transferring” a certain amount of personal privacy, consumers can, on the one hand, receive personalized recommendations or intelligent advertisements by e-commerce platforms, which could reduce item search costs and improve shopping efficiency, and thus obtain extra benefits. On the other hand, transferring personal information may also lead to spam and other problems, resulting in losses. Based on the above analysis, the following assumptions concerning the profits of consumers with third-party regulators are made:

H1: *B*_1_ (*B*_1_ > 0) represents the fixed benefits obtained by consumers who choose the strategy of “not sharing” when they shop online, and *B*_2_ (*B*_2_ > 0) represents the fixed benefits obtained by consumers who choose the strategy of “sharing” when they shop online. We assume that *B*_2_ > *B*_1_.
H2: *N*_1_ (*N*_1_ > 0) is the maximum extra benefits generated for consumers by the data mining behavior of e-commerce platforms. The positive utility coefficient of data mining for consumers is α (α > 0). Thus, the extra benefits brought by data mining are α*N*_1_.
H3: *N*_2_ (*N*_2_ > 0) is the maximum extra losses brought to consumers by the data mining behavior of e-commerce platforms. The negative utility coefficient of data mining for consumers is β (β < 0). Thus, the extra losses brought by data mining are β*N*_2_.
H4: Y (*Y* ≥ 0) is the reward obtained by consumers from e-commerce platforms for sharing personal information (such as high-level membership rights, coupons, etc.).

#### E-Commerce Platforms

When consumers register an account to engage in online shopping on e-commerce platforms, regardless of whether they choose to share personal information, e-commerce platforms can obtain fixed benefits through the expansion of user scale, increased transaction volume and the enhancement of attractiveness to brand advertisers. Moreover, e-commerce platforms can also obtain extra benefits. The application of AI technologies in personal data mining and consumer behavior analysis will help platforms achieve precision marketing and provide personalized decision support, which can improve recommendation quality and promote marketing transformation. Based on the above analysis, hypothesis 5 and hypothesis 6 are proposed:

H5: *F* (*F* > 0) represents the fixed benefits obtained by e-commerce platforms when providing services to consumers, *M*_1_ (*M*_1_ > 0) is the maximum extra benefits that e-commerce platforms can obtain from consumers through data mining when the latter choose the strategy of “sharing”; *M*_2_ (0 < *M*_2_ < *M*_1_) is the maximum extra benefit obtained by e-commerce platforms from consumers through illegal data mining when the latter choose the strategy of “not sharing.”
H6: μ (0 < μ < 1) is the ability of e-commerce platforms to apply AI technologies. The value of μ is positively related to the ability to mine data and the efficiency of precision marketing. The higher the value is, the greater the extra benefits that will be obtained by e-commerce platforms.

With third-party regulatory agencies supervising the compliance of the data mining behavior of e-commerce platforms, consumers are more likely to share personal information out of trust in e-commerce platforms, and e-commerce platforms will receive extra benefits, which can be considered trust benefits. If e-commerce platforms mine the personal information of consumers when the latter choose the “not sharing” strategy, illegal behavior may be detected by third-party regulators. In this case, e-commerce platforms would lose the trust benefits of consumers and thus suffer extra losses. In addition, these platforms would be punished by third-party regulators. Based on the above analysis, hypotheses 7 and 8 are proposed.

H7: The possibility that the illegal data mining behavior of e-commerce platforms is discovered when consumers choose the behavior strategy of “not sharing” is δ (0 ≤ δ ≤ 1), which reflects the supervision level of third-party regulators. The penalty for illegal data mining on e-commerce platforms is *f*.
H8: *t* is the trust coefficient of consumers for the e-commerce platform under the supervision of third-party regulatory agencies. Then, δ * *tM*_1_ represents the trust benefits obtained by e-commerce platforms, and these benefits will be lost when illegal data mining behavior is detected by regulatory agencies.
H9: *C*_1_ (*C*_1_ = *Y*) is the reward cost of e-commerce platforms when consumers choose to share personal information; *C*_2_ (*C*_2_ > 0) is the technical cost of data mining for e-commerce platforms, such as the cost of information maintenance and information processing.
H10: Consumers choose to share personal information with probability *p* (0 ≤ *p* ≤ 1), and the probability of consumers not sharing personal information is 1 − *p*. E-commerce platforms choose data mining with probability *q* (0 ≤ *q* ≤ 1) and not to mine with probability 1 − *q*.

Based on the hypotheses above, the notations for the model are displayed in Table 1, and the payoff matrix involving consumers and e-commerce platforms is shown in Table 2.

**Table 1:** Notations for the model.

**Notations**	**Descriptions**
*B*_1_	The fixed profits obtained by consumers when they choose not to share personal information when online shopping
*B*_2_	The fixed profits obtained by consumers when they choose to share personal information when online shopping
*N*_1_	Maximum extra benefits generated for consumers by the data mining behavior of e-commerce platforms
α	The positive utility coefficient of data mining for consumers
*N*_2_	Maximum extra losses generated for consumers by the data mining behavior of e-commerce platforms.
β	The negative utility coefficient of data mining for consumers
*Y*	Rewards obtained by consumers from e-commerce platforms for sharing personal information
*F*	The fixed benefits obtained by e-commerce platforms when providing services to consumers.
*M*_1_	The maximum extra benefits that e-commerce platforms can obtain through data mining when consumers choose to share personal information.
*M*_2_	The maximum extra benefits obtained by e-commerce platforms through illegal data mining when consumers choose not to share personal information.
μ	The ability of e-commerce platforms to use AI technologies, which directly influences the effects of data mining.
δ	The possibility that illegal data mining behavior by e-commerce platforms is detected when consumers choose not to share personal information.
*f*	The penalty for illegal data mining by e-commerce platforms.
*t*	The trust coefficient of consumers for e-commerce platforms under the supervision of third-party regulators
*C*_1_	The reward cost of e-commerce platforms when consumers choose to share personal information
*C*_2_	The technical cost of data mining for e-commerce platforms
*p*	Probability of consumers choosing to share personal information
*q*	Probability of e-commerce platforms choosing data mining

**Table 2:** Payoff matrix of consumers and e-commerce platforms in privacy protection.

		**E-commerce platforms**	
	**Strategy**	**Data mining (q)**	**Not data mining (1 − *q*)**
Consumers	Sharing (*p*)	(*B*_2_ + α*N*_1_ + β*N*_2_ + *Y*, $\begin{array}{l} F+\mu M_{1}+\delta^{*}tM_{1}-C_{1}-C_{2}) \end{array}$	(*B*_2_ + *Y*,*F* − *C*_1_)
	Not sharing (1 − *p*)	(*B*_1_ + α*N*_1_ + β*N*_2_,$F+\mu M_{2}-\delta^{*}tM_{1}-\delta f-C_{2}$)	(*B*_1_,*F*)

## Evolutionary Game Analysis

### Equilibrium Points of the Evolutionary Game

The expected profits of “sharing” and “not sharing” are *E*_1*y*_ and *E*_1*n*_, respectively. The average profit is $\bar{E_{1}}$. Then,

(1) $$\begin{array}{l} E_{1y}=q\left(B_{2}+\alpha N_{1}+\beta N_{2}+Y\right)+\left(1-q\right)\left(B_{2}+Y\right) \end{array}$$

(2) $$\begin{array}{l} E_{1n}=q\left(B_{1}+\alpha N_{1}+\beta N_{2}\right)+\left(1-q\right)B_{1} \end{array}$$

(3) $$\begin{array}{l} \bar{E_{1}}=qE_{1y}+\left(1-q\right)E_{1n} \end{array}$$

The replicator dynamic equation of consumers is as follows:

(4) $$\begin{array}{l} F\left(p\right)=\frac{dp}{dt}=p\left(E_{1y}-\bar{E_{1}}\right)=p^{*}{\left(1-p\right)}^{*}\left(B_{2}-B_{1}+Y\right) \end{array}$$

The expected profits when e-commerce platforms choose “data mining” and “not data mining” are *E*_2*y*_ and *E*_2*n*_, respectively. The average profit is $\bar{E_{2}}$. Then,

(5) $$\begin{array}{l} E_{2y}=p(F+\mu M_{1}+\delta^{*}tM_{1}-C_{1}-C_{2})+(1-p)(F\\ \;+\mu M_{2}-\delta^{*}tM_{1}-\delta f-C_{2}) \end{array}$$

(6) $$\begin{array}{l} E_{2n}=p\left(F-C_{1}\right)+\left(1-p\right)F \end{array}$$

(7) $$\begin{array}{l} \bar{E_{2}}=pE_{2y}+\left(1-p\right)E_{2n} \end{array}$$

The replicator dynamic equation of e-commerce platforms is as follows:

(8) $$\begin{array}{l} F(q)=\frac{d_{q(t)}}{d_{t}}=q(1-q)[\mu M_{2}-\delta^{*}tM_{1}-\delta f-C_{2}\\ \;+(\mu M_{1}+2^{*}\delta tM_{1}-\mu M_{2}+\delta f)p] \end{array}$$

According to Equations (4) and (8), the replicator dynamic equations for a two-player game of privacy protection between consumers and e-commerce platforms are

(9) $$\begin{array}{l} \{\begin{array}{l} \frac{d_{p(t)}}{d_{t}}=p(E_{1y}-\bar{E_{1}})=p(1-p)(B_{2}-B_{1}+Y)\\ \frac{d_{q(t)}}{d_{t}}=q(E_{2y}-\bar{E_{2}})=q(1-q)[\mu M_{2}-\delta^{*}tM_{1}-\delta f-C_{2}\\ +(\mu M_{1}+2^{*}\delta tM_{1}-\mu M_{2}+\delta f)p] \end{array} \end{array}$$

Let *F*(*p*) = 0 and *F*(*q*) = 0, and the equilibrium point of the evolutionary game can be obtained by solving Equation (10):

(10) $$\begin{array}{l} \{\begin{array}{l} p(1-p)(B_{2}-B_{1}+Y)=0\\ q(1-q)[\mu M_{2}-\delta^{*}tM_{1}-\delta f-C_{2}+(\mu M_{1}+2^{*}\delta tM_{1}\\ -\mu M_{2}+\delta f)p]=0 \end{array} \end{array}$$

The equilibrium points are *E*_1_ = (0, 0), *E*_2_ = (0, 1), *E*_3_ = (1, 0), *E*_4_ = (1, 1).

### Stability of Equilibrium Points

According to Friedman (42), the stability of equilibrium points can be deduced by analyzing the local stability of the Jacobian matrix. The Jacobian matrix is expressed as $J=\left[\begin{array}{c} \frac{\partial F\left(p\right)}{\partial p}\\ \frac{\partial F\left(q\right)}{\partial p} \end{array}\begin{array}{c} \frac{\partial F\left(p\right)}{\partial q}\\ \frac{\partial F\left(q\right)}{\partial q} \end{array}\right]=\left(\begin{array}{c} a_{11}\\ a_{21} \end{array}\begin{array}{c} a_{12}\\ a_{22} \end{array}\right)$, in which *a*_11_ = (1 − 2*p*)(*B*_2_ − *B*_1_ + *Y*), *a*_12_ = 0, $a_{21}=q\left(1-q\right)\left(\mu M_{1}+2^{*}\delta tM_{1}-\mu M_{2}+\delta f\right)$, and $a_{22}=\left(1-2q\right)\left[\mu M_{2}-\delta^{*}tM_{1}-\delta f-C_{2}+\left(\mu M_{1}+2^{*}\delta tM_{1}-\mu M_{2}+\delta f\right)p\right]$.

The equilibrium point will be the evolutionary stable strategy when:


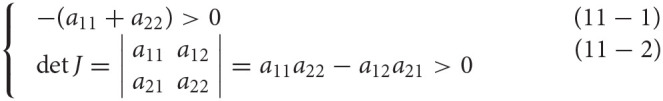


At $E_{1}=(0,0),\;\det J=\left[\begin{array}{cc} B_{2}-B_{1}+Y & 0\\ 0 & \mu M_{2}-\delta tM_{1}-\delta f-C_{2} \end{array}\right]$. *a*_11_ = *B*_2_ − *B*_1_+*Y* > 0, and formula (11-1) would be satisfied when *a*_22_ < 0, while formula (11-2) can be satisfied by requiring *a*_22_ > 0, which is impossible. Thus, *E*_1_ is unstable.

At $E_{2}=(0,1),\;\det J=\left[\begin{array}{cc} B_{2}-B_{1}+Y & 0\\ 0 & C_{2}+\delta f+\delta tM_{1}-\mu M_{2} \end{array}\right]$. *a*_11_ = *B*_2_ − *B*_1_+*Y* > 0, and formula (11-1) would be satisfied when *a*_22_ < 0, while formula (11-2) can be satisfied when *a*_22_ > 0, which is impossible. Thus, *E*_2_ is unstable.

At $E_{3}=(1,0),\;\det J=\left[\begin{array}{cc} B_{1}-B_{2}-Y & 0\\ 0 & \mu M_{1}+\delta^{*}tM_{1}-C_{2} \end{array}\right]$. *B*_1_ − *B*_2_ − *Y* < 0, and when $\mu M_{1}+\delta^{*}tM_{1}-C_{2}<0$, *E*_3_ = (1, 0) is stable. This indicates that when the sum of fixed benefits and reward benefits obtained by consumers due to sharing personal information (*B*_2_ + *Y*) is greater than the fixed benefits of not sharing personal information (*B*_1_) and when the extra benefits including consumer trust and application ability of AI technologies ($\mu M_{1}+\delta^{*}tM_{1}$) are less than the technical cost of data mining (*C*_2_), the evolutionary stable strategy will be (1, 0); that is, consumers choose to share personal information when *p* = 1, and e-commerce platforms do not carry out data mining when *q* = 0.

At $E_{4}=(1,1),\;\det J=\left[\begin{array}{cc} B_{1}-B_{2}-Y & 0\\ 0 & C_{2}-\mu M_{1}-\delta^{*}tM_{1} \end{array}\right]$. *B*_1_ − *B*_2_ − *Y* < 0, and when $C_{2}-\mu M_{1}-\delta^{*}tM_{1}<0$, *E*_4_ = (1, 1) is the stable equilibrium point. This shows that when the sum of fixed benefits and reward benefits obtained by consumers from sharing personal information (*B*_2_ + *Y*) is greater than the fixed benefits of not sharing personal information (*B*_1_) and when the extra benefits coming from consumer trust and the applicability of AI technologies ($\mu M_{1}+\delta^{*}tM_{1}$) are greater than the technical cost of data mining (*C*_2_), the evolutionary stable strategy will be (1, 1); that is, consumers choose the strategy of “sharing” when *p* = 1, and e-commerce platforms choose data mining when *q* = 1.

**Figure 1:**
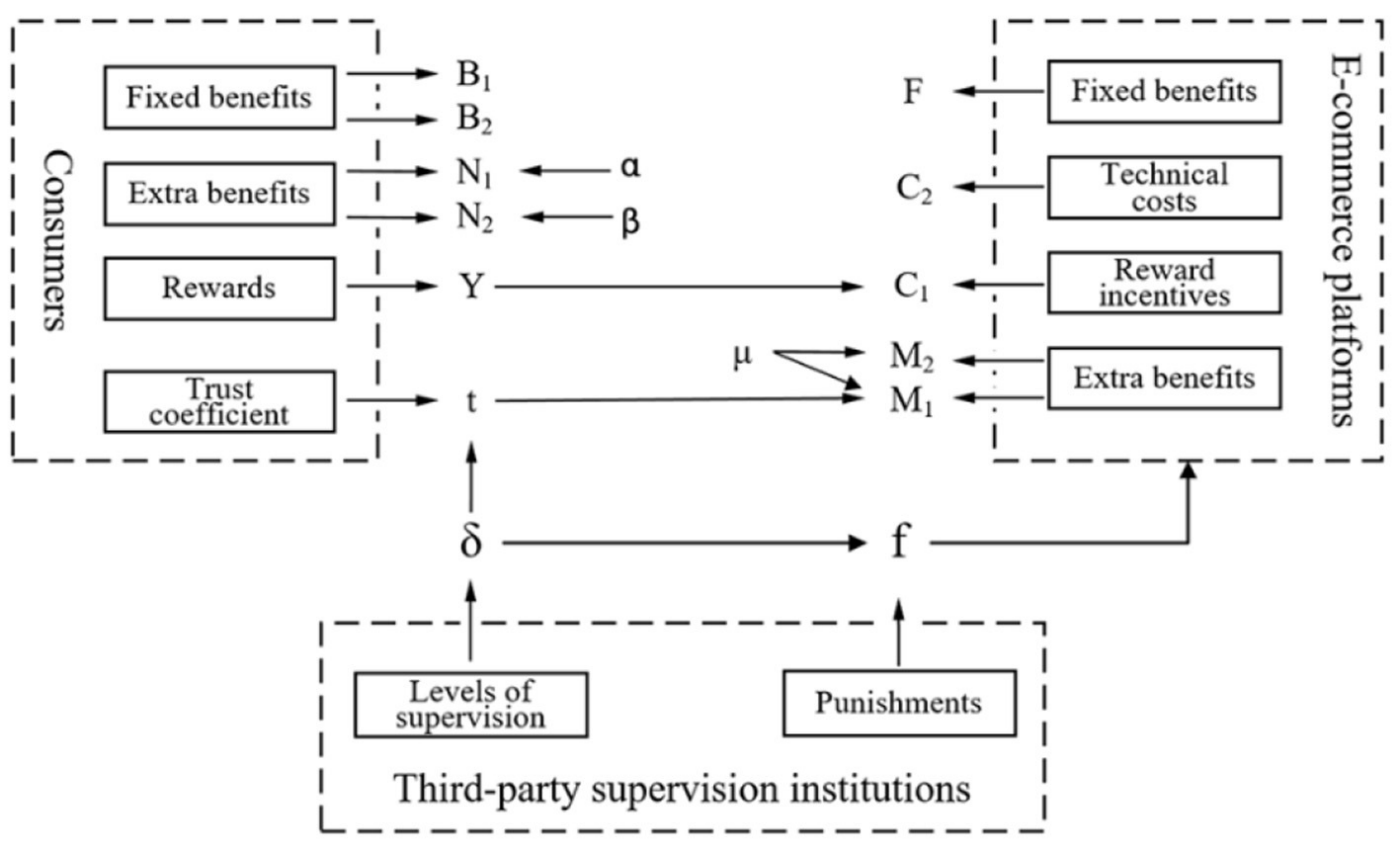
Influencing factors and their relevance.

Based on the results, the influencing factors of the strategy selection of e-commerce platforms and consumers are illustrated in Figure 1. From the perspective of consumers, the fixed benefits of online shopping and rewards create incentives to share personal information, and the extra benefits related to the benefit and loss coefficient will not affect consumers' strategy selection. From the perspective of e-commerce platforms, extra benefits, which could be adjusted by consumers' trust and the applicability of AI technologies, are crucial for data mining. Additionally, the technical cost of data mining should be taken into consideration since an increase in the technical cost will prevent e-commerce platforms from expanding their profits, while the reward cost has no impact. Note that the penalty has no influence on e-commerce platforms' data mining behavior, while the supervision level plays an important role since it affects the extra benefits by influencing consumer trust. This may because e-commerce platforms' data mining practice changes frequently in light of technological advance in AI, and thus it is challenging for third-party supervision organizations to find comprehensive and updated information firm by firm and accordingly impose penalties. Another reason might be the lags in direct regulation on e-commerce platforms' data mining action. It is difficult to ensure that the regulation is updated with every round of technological advance, which makes penalty infeasible. Therefore, improving supervision level to promote industry self-regulation could be feasible and effective for privacy protection. E-commerce platforms know more about AI technology and data practice, and therefore are better positioned to identify best practices (8). This suggests that the supervision of third-party regulators could be complementary to industry attempts to self-regulate.

Conclusions could be initially drawn that (1) the improvement of online shopping benefits due to the advancements of AI technologies could fundamentally encourage consumers to share personal information; (2) e-commerce platforms are supposed to provide various and generous reward incentives to consumers to encouraging the sharing of personal information, since the reward cost does not decrease the profits of data mining; (3) it is profitable for e-commerce platforms to use AI technologies to determine people's shopping habits, but they should make efforts to reduce the cost of technology adoption; and (4) when the probability of the illegal data mining being discovered is large enough, third-party supervision could serve as a strong deterrent for e-commerce platforms, which can effectively enable consumers to trust e-commerce platforms and share personal information.

## Simulation Results and Analysis

To more intuitively reflect the evolutionary game process and the evolutionary stability equilibrium strategy between consumers and e-commerce platforms in different situations, a simulation analysis of the stability strategy results of the two subjects under different parameters was carried out using MATLAB. The parameters were set based on model assumptions that are in line with economic reality. As for parameter settings of consumers, the value of Y is set to be no larger than that of *B*_1_ to ensure consumers to share personal information. In terms of parameter settings of e-commerce platforms, the value of *f* is supposed to be smaller than that of *F* so as to ensure the operation of the e-commerce platform; the value of *C*_1_ + *C*_2_ should not exceed the sum of *F* and *M*_2_. Moreover, the parameter of α, β, δ, *t*, and μ are set to be 0.5 to maintain neutrality. Although few simulation experiments have been conducted to investigate privacy protection in the context of online shopping against the background of AI technology advancements, parameter settings in other fields, such as online advertising (43), are available for reference. The specific parameter settings are listed in Tables 3, 4.

**Table 3:** Parameter settings of consumers.

**Parameters**	***B*_1_**	***B*_2_**	***N*_1_**	***N*_2_**	**α**	**β**	**Y**
Value	5	10	5	5	0.5	−0.5	5

**Table 4:** Parameter settings of e-commerce platforms.

**Parameters**	***F***	***f***	***M*_1_**	***M*_2_**	**μ**	**δ**	***t***	***C*_1_**	***C*_2_**
Value	5	5	10	5	0.5	0.5	0.5	5	2

**Figure 2:**
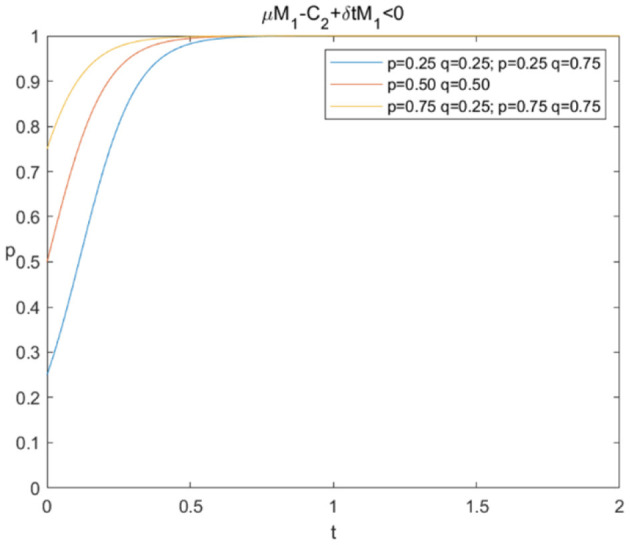
Evolutionary stability strategy of consumers when $\mu M_{1}+\delta^{*}tM_{1}-C_{2}<0$.

**Figure 3:**
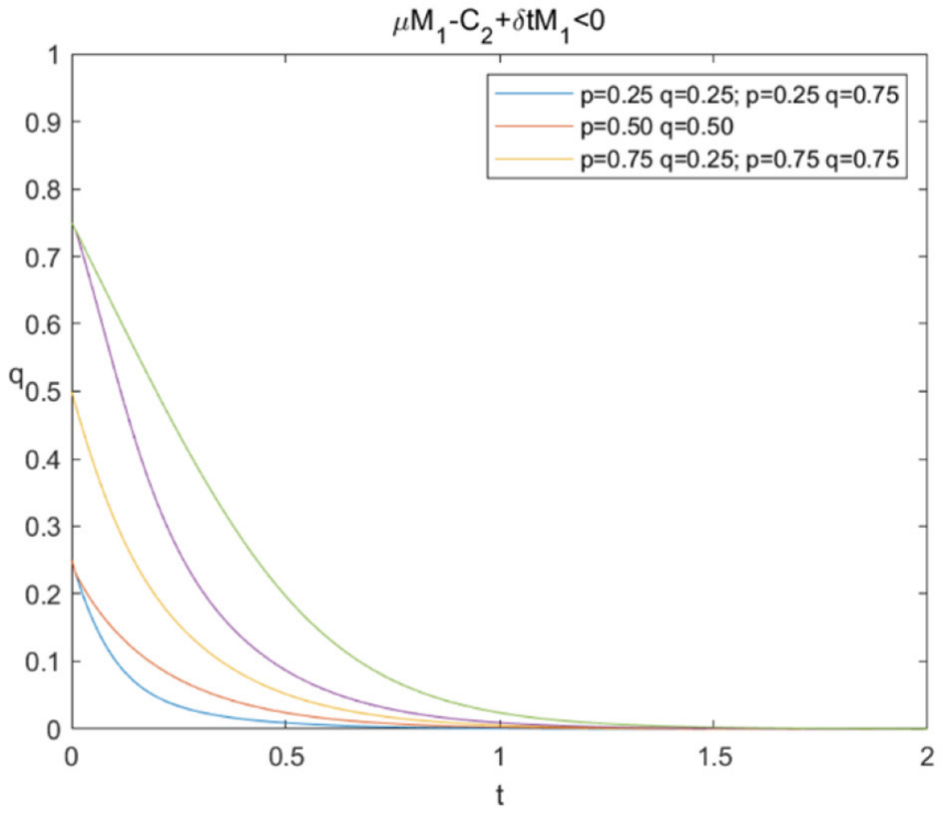
Evolutionary stability strategy of e-commerce platforms when $\mu M_{1}+\delta^{*}tM_{1}-C_{2}<0$.

The evolutionary stability strategy of consumers and e-commerce platforms when $\mu M_{1}+\delta^{*}tM_{1}-C_{2}<0$ is shown in Figures 2, 3, respectively.

Figure 2 shows that when $\mu M_{1}+\delta^{*}tM_{1}-C_{2}<0$, the probability of sharing personal information increases rapidly under different circumstances, and p^*^ = 1 is the evolutionary stability strategy. This indicates that consumers will ultimately choose to share personal information. In Figure 3, the probabilities of data mining decline rapidly under different circumstances, and q^*^ = 0 is the evolutionary stability strategy, which means that e-commerce platforms prefer “not mining.” The simulation results confirm that if e-commerce platforms have no extra benefits after deducting the technical cost of data mining, they will not choose to conduct data mining even if consumers share personal information.

**Figure 4:**
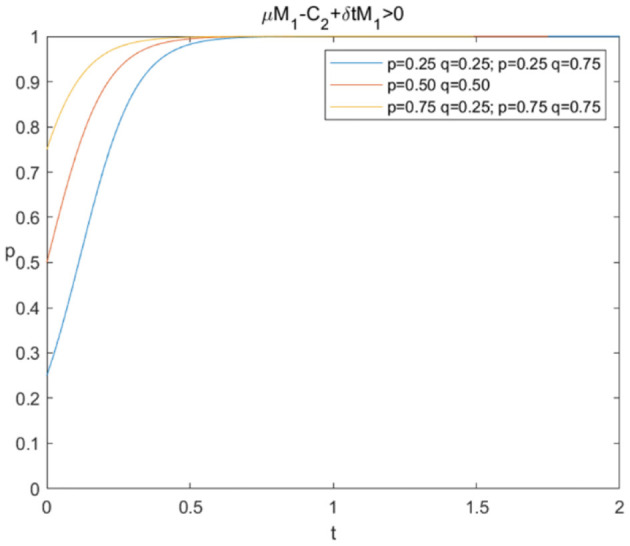
Evolutionary stability strategy of consumers when $\mu M_{1}+\delta^{*}tM_{1}-C_{2}>0$.

**Figure 5:**
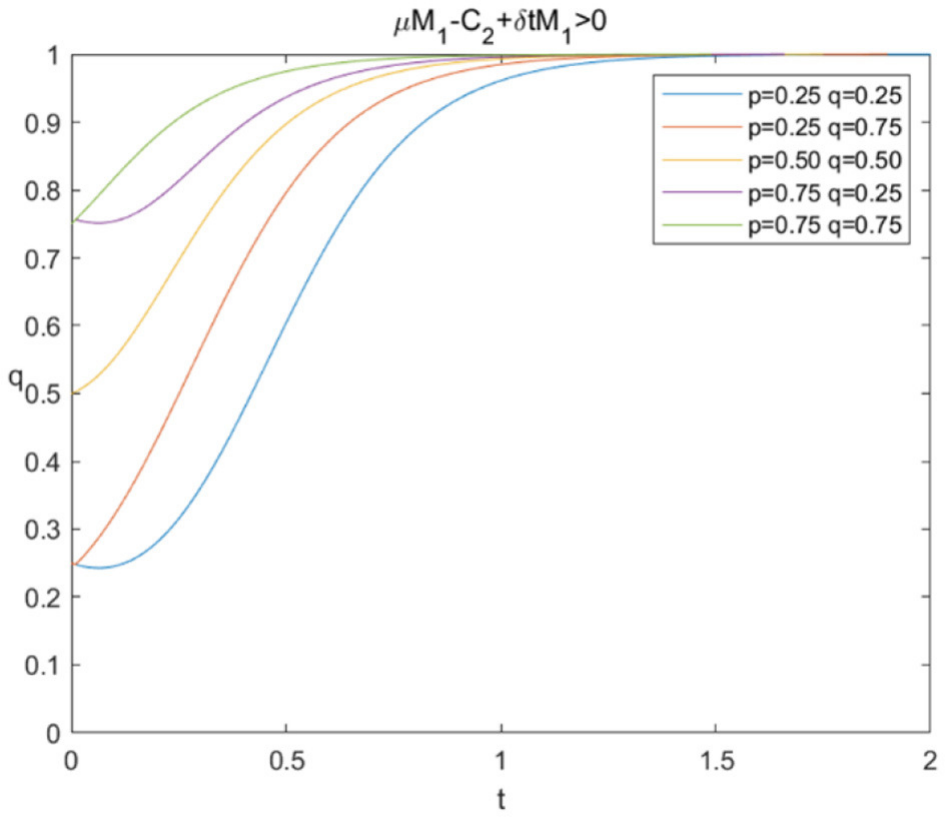
Evolutionary stability strategy of e-commerce platforms when $\mu M_{1}+\delta^{*}tM_{1}-C_{2}>0$.

The evolutionary stability strategy of consumers and e-commerce platforms when $\mu M_{1}+\delta^{*}tM_{1}-C_{2}>0$ is shown in Figures 4, 5, respectively.

Figure 4 illustrates that when $\mu M_{1}+\delta^{*}tM_{1}-C_{2}>0$, the probability of sharing personal information increases rapidly under different circumstances, and p^*^ = 1 is the evolutionary stability strategy. Figure 5 shows that the probabilities of data mining rise quickly to the value of 1 under different circumstances, which means that q^*^ = 1 is the evolutionary stability strategy and e-commerce platforms would prefer “mining.” The simulation results verify that e-commerce platforms can increase extra benefits through AI technologies and consumer trust or reduce technical costs to achieve the result of data mining when consumers choose to share personal information, and the model can eventually evolve toward a win-win situation.

## Discussion

In the context of AI technology empowering the retail industry, especially e-commerce, it will become normal for consumers to actively share personal information. Based on the results of this study, some suggestions are proposed to achieve a win-win situation with benefit maximization for both consumers and e-commerce platforms under the condition that consumers' privacy is well protected.

For e-commerce platforms, effectively using AI technologies at a lower cost will be more profitable when mining the personal data of consumers. Therefore, e-commerce platforms should make efforts to adopt AI technologies to improve the quality of recommendations, for instance, providing personalized information services and decision support based on recommendation system algorithms and deep learning technologies. In addition, accurate advertising and AI video marketing can be achieved with the application of video structuring and image retrieval technology in addition to machine learning, deep learning, and computer vision techniques. Furthermore, it is important to reduce the cost of using AI technologies. Purchasing AI services in the form of cloud services or software can be combined with independent research and development. Since consumer trust will influence the profits of data mining, e-commerce platforms need to protect consumers' privacy through a combination of appropriate control, security, transparency and consent mechanisms related to the collection and use of their personal data (2) to increase consumer trust, and privacy policy statements should be actively provided and updated in a timely manner to increase the possibility of consumers sharing personal information. For example, e-commerce platforms are supposed to thoroughly describe their internal guidelines on procedural, organizational and technical requirements for the collection, storage and processing of personal data.

Results show that consumers are willing to share personal information without the influence of the extra benefits brought by data mining when they are shopping online. This indicates that data use is likely to grow with data mining technology such as AI in the future. However, it is important to be aware that sophisticated consumers may anticipate the uncertainty of illegal data mining and hesitate to give away personal data. They would trade off between immediate gains from the online transaction and potential loss from future data use. Some people argue that data mining and future data use are new attributes of online shopping, and e-commerce platforms in a competitive market will respect consumers' preference for limited data mining and use as long as the attributes are clearly conveyed between consumers and e-commerce platforms via a well-written privacy policy (8). However, these attributes are not currently well-defined at the time of online shopping, and they can evolve over time in ways that depend on the e-commerce platform's data policy but are completely out of consumers' control, ability to predict or ability to value. Therefore, consumers need to enhance their awareness of privacy protection despite the incentives for sharing personal information being attractive. According to McDonald and Cranor (44), most consumers do not read privacy notices. First, consumers should be aware of inadequate privacy protection by e-commerce platforms. Access to personal information should be allowed after the perusal of privacy notices provided by e-commerce platforms. Additionally, consumers are expected to realize that the actual data practices of some firms will deviate from the privacy notice they disclose. Therefore, they need to avoid the excessive transfer of personal information for the purpose of rewards and reduce unnecessary personal information sharing.

From the perspective of third-party supervision institutions, professional competence should be improved, and supervision should be enhanced through digital tools so that illegal data mining by e-commerce platforms can be detected in a timely manner. It is necessary for regulators to focus on whether e-commerce platforms guarantee data security on the basis of providing sufficient information to consumers since most data security practices are not visible until someone exposes the data vulnerability. For example, a rating system on data practices could be developed to monitor e-commerce platforms. Moreover, accountability systems could be established. Blockchains could be adopted to track every piece of data and develop AI to predict the likelihood of every adverse event. Last, governments should encourage responsible data practices and foster consumer-friendly innovations.

Referring to future research, regulators will play more important roles in consumer privacy protection with the rapid advancements of AI technologies and their wide application in the e-commerce industry. The costs and benefits of supervision could be further analyzed, and a three-party evolutionary game model could be structured to investigate the influencing mechanisms in the future.

## Conclusions

This work studies the strategy selections of consumers and e-commerce platforms in privacy protection based on evolutionary game theory. The results show that the privacy protection strategies of consumers and e-commerce platforms are closely related to the benefits they could obtain and that the model can eventually evolve toward a win-win situation. Based on the analysis, the following conclusions can be drawn.

First, the application of AI technologies to e-commerce will fundamentally change the consumption habits of customers. It is normal for consumers to actively share personal information when they shop online since the fixed benefit is the only decisive factor and generous rewards are incentives to share personal information.

Second, it is profitable for e-commerce platforms to mine consumer data by improving the ability to use AI technologies and making efforts to lower the technical cost. The evolution game will evolve toward a win-win situation as long as e-platforms can maintain positive earnings by adjusting the extra benefits and technical costs of data mining.

Third, third-party supervision will bring about differences in consumer trust, which influences the extra benefits of e-commerce platforms. In this case, the government should improve the level of supervision instead of imposing a high penalty to enhance consumer trust, which could protect consumers' privacy.

## Data Availability Statement

The original contributions presented in the study are included in the article/supplementary material, further inquiries can be directed to the corresponding author/s.

## Author Contributions

SW, YX, and CL contributed to conception and design of the study. SW performed the statistical analysis and drafted the manuscript. ZC and YX revised the work critically for important intellectual content. ZC agreed to be accountable for all aspects of the work in ensuring that questions related to the accuracy or integrity of any part of the work are appropriately investigated and resolved. All authors contributed to the article and approved the submitted version.

## Conflict of Interest

The authors declare that the research was conducted in the absence of any commercial or financial relationships that could be construed as a potential conflict of interest.

## References

[1] Guthrie C Fosso-Wamba S Arnaud JB. Online consumer resilience during a pandemic: an exploratory study of e-commerce behavior before, during and after a COVID-19 lockdown. J Retail Consum Serv. (2021) 61:102570. 10.1016/j.jretconser.2021.102570

[2] Netcomm Suisse Observatory United Nations Conference on Trade and Development (UNCTAD). COVID-19 and E-COMMERCE Findings From Survey of Online Consumers in 9 Countries. (2020). Available online at: https://digitallibrary.un.org/record/3886558 (accessed June 21, 2021).

[3] Pantano E Pizzi G Scarpi D Dennis C. Competing during a pandemic? Retailers' ups downs during the COVID-19 outbreak. J Bus Res. (2020) 116:209–13. 10.1016/j.jbusres.2020.05.036 32501307 PMC7241368

[4] Sheth J. Impact of Covid-19 on consumer behavior: will the old habits return or die? J Bus Res. (2020) 117:280–3. 10.1016/j.jbusres.2020.05.059 32536735 PMC7269931

[5] Deloitte. COVID-19 Will Permanently Change E-commerce in Denmark. (2020). Available online at: https://www2.deloitte.com/content/dam/Deloitte/dk/Documents/strategy/e-commerce-covid-19-onepage.pdf (accessed June 21, 2021).

[6] Abdelrhim M Elsayed A. The effect of COVID-19 spread on the E-commerce market: the case of the 5 largest E-commerce companies in the world. Social Science Research Network. (2020). 10.2139/SSRN.3621166

[7] Mazurek G Małagocka K. Perception of privacy and data protection in the context of the development of artificial intelligence. J Manag Anal. (2019) 6:344–64 10.1080/23270012.2019.1671243

[8] Jin GZ. Artificial intelligence and consumer privacy. In: Working Paper 24253. (2018). Available online at: http://www.nber.org/papers/w24253 (accessed June 21, 2021).

[9] WTO (World Trade Organization). E-commerce, Trade and the COVID-19 Pandemic. (2020). Available online at: https://www.wto.org/english//tratop_e/covid19_e/ecommerce_report_e.pdf (accessed June 21, 2021).

[10] Dinev T Hart P. Internet privacy concerns and social awareness as determinants of intention to transact. Int J Electron Commer. (2005) 10:7–29. 10.2753/JEC1086-4415100201

[11] Castañeda JA Montoro FJ. The effect of Internet general privacy concern on customer behavior. Electron Commerce Res. (2007) 7:117–41. 10.1007/s10660-007-9000-y

[12] Pew Research Center. The State of Privacy in Post-Snowden America. (2016). Available online at: http://www.pewresearch.org/fact-tank/2016/09/21/the-state-of-privacy-in-america/ (accessed June 21, 2021).

[13] Ovaska S. Data Privacy Risks to Consider When Using AI. Financial Management (2020). Available online at: https://www.fm-magazine.com/issues/2020/feb/data-privacy-risks-when-using-artificial-intelligence.html (accessed June 21, 2021).

[14] Pantziou PBG. A k-anonymity privacy-preserving approach in wireless medical monitoring environments. Pers Ubiquitous Comput. (2014) 18:61–74. 10.1007/s00779-012-0618-y

[15] Yang S Yin G Ma C. A random anonymity framework for location privacy. Wuhan Univ J Nat Sci. (2015) 20:521–9. 10.1007/s11859-015-1128-3

[16] Vergara-Laurens IJ Mendez D Jaimes LG Labrador M. A-PIE: an algorithm for preserving privacy, quality of information, and energy consumption in participatory sensing systems. Pervasive Mob Comput. (2016) 32:93–112. 10.1016/j.pmcj.2016.06.020

[17] Lu R Lin X Shen X. SPOC: a secure and privacy-preserving opportunistic computing framework for mobile-healthcare emergency. IEEE Transact Parallel Distribut Syst. (2013) 24:614–24. 10.1109/TPDS.2012.14624018958

[18] Li Y Li Y Yan Q Deng RH Privacy leakage analysis in online social networks. Comput Secur. (2015) 49:239–54. 10.1016/j.cose.2014.10.012

[19] Wu Y Pan L. SG-PAC: a stochastic game approach to generate personal privacy paradox access-control policies in social networks. Comput Secur. (2020) 102:102157. 10.1016/j.cose.2020.102157

[20] Chen R Fung BC Yu PS Desai BC. Correlated network data publication via differential privacy. VLDB J. (2014) 23:653–76. 10.1007/s00778-013-0344-8

[21] Li C Hay M Miklau G Wang Y. A data-and workload-aware algorithm for range queries under differential privacy. Proc VLDB Endow. (2014) 7:341–52. 10.14778/2732269.2732271

[22] Li K Tian L Li W Luo G Cai Z. Incorporating social interaction into three-party game towards privacy protection in IoT. Comput Netw. (2019) 150:90–101. 10.1016/j.comnet.2018.11.036

[23] Squicciarin AC Shehab M Wede J. Privacy policies for shared content in social network sites. LDB J. (2010) 19:777–96. 10.1007/s00778-010-0193-7

[24] Wang W Yang L Chen Y Zhang Q. A privacy-aware framework for targeted advertising. Comput Netw. (2015) 79:17–29. 10.1016/j.comnet.2014.12.017

[25] Kumari V Chakravarthy S. Cooperative privacy game: a novel strategy for preserving privacy in data publishing. Hum Cent Comput Inf Sci. (2016) 6:12. 10.1186/s13673-016-0069-y

[26] Mengibaev U Jia X Ma Y. The impact of interactive dependence on privacy protection behavior based on evolutionary game. Appl Math Comput. (2020) 379:125231. 10.1016/j.amc.2020.125231

[27] Li Y Xu L Liu B. Evolutionary game analysis on e-commerce personalization and privacy protection. Wuhan Univ J Nat Sci. (2018) 23:17–24. 10.1007/s11859-018-1289-y

[28] Sun PJ. The optimal privacy strategy of cloud service based on evolutionary game. Cluster Comput. (2020) 10.1007/s10586-020-03164-5

[29] Phelps J Nowak G Ferrell E. Privacy concerns and consumer willingness to provide personal information. J Public Policy Mark. (2000) 19:27–41. 10.1509/jppm.19.1.27.16941

[30] Claypool M Le P Waseda M Brown D. Implicit interest indicators. In: Proc. of 6th Conference on Intelligent User Interfaces Santa Fe, NM (2002). p. 33–40.

[31] Kim H Chan P. Learning implicit user interest hierarchy for context in personalization. In: Proc. of the International Conference on Intelligent User Interfaces. Miami, FL (2003). p. 101–8.

[32] Joachims T Granka L Pan B Hembrooke H Gay G. Accurately interpreting click-through data as implicit feedback. In: Proc. of the 17th Annual International ACM SIGIR Conference. Salvador (205). p. 154–61.

[33] Bobadilla J Ortega F Hernando A Gutiérrez A Recommender systems survey. Knowledge-Based Syst. (2013) 46:109–32. 10.1016/j.knosys.2013.03.012

[34] Earp JB Baumer D. Innovative web use to learn about consumer behavior and online privacy. Commun ACM. (2003) 46:81–3. 10.1145/641205.641209

[35] White TB. Consumer disclosure and disclosure avoidance: a motivational framework. J Consum Psychol. (2004) 14:41–51. 10.1207/s15327663jcp1401&amp;2_6

[36] Athey S Catalini C Tucker CE. The digital privacy paradox: Small money, small costs, small Talk. In: MIT Sloan Research Paper No. 5196-17, Stanford University Graduate School of Business Research Paper No. 17-14. (2017). Available online at: https://ssrn.com/abstract=2916489 (accessed June 21, 2021).

[37] Sun Y Fang S Hwang Y. Investigating privacy and information disclosure behavior in social electronic commerce. Sustainability. (2019) 11:3311. 10.3390/su11123311

[38] Fazal-E-Hasan SM Ahmadi H Mortimer G Lings I Kelly L Kim H. Online repurchasing: the role of information disclosure, hope, and goal attainment. J Consum Aff. (2020) 54:198–226. 10.1111/joca.12263

[39] Zeng F Ye Q Li J Yang Z. Does self-disclosure matter? A dynamic two- stage perspective for the personalization-privacy paradox. J Bus Res. (2020) 124:667–75. 10.1016/j.jbusres.2020.02.006

[40] Kobsa A. Privacy-enhanced web personalization. In: Brusilovsky P Kobsa A Nejdl W editors. The Adaptive Web. Lecture Notes in Computer Science, Vol. 4321. Berlin, Heidelberg: Springer (2007).

[41] Fung A Graham M Weil D. Full Disclosure: The Perils and Promise of Transparency. Cambridge: Cambridge University Press (2007).

[42] Friedman D. Evolutionary game in economics. Econometrica. (1991) 59:637–66. 10.2307/2938222

[43] He X Li L Zhang H Zhu X. Social media or website? Research on online advertising type based on evolutionary game. In: Lang KR Xu J Zhu B Liu X Shaw MJ Zhang H Fan M editors. Smart Business: Technology and Data Enabled Innovative Business Models and Practices. WeB 2019. Lecture Notes in Business Information Processing, Vol. 403. Cham: Springer (2020).

[44] McDonald AM Cranor L. The cost of Reading Privacy Policies. (2009). Available online at: https://www.semanticscholar.org/paper/The-Cost-of-Reading-Privacy-Policies-McDonald-Cranor/7b5fb5ee261d7be84712eba85e786b4e4042fd2e (accessed June 21, 2021).

